# Visual Performance of Children with Amblyopia after 6 Weeks of Home-Based Dichoptic Visual Training

**DOI:** 10.3390/children11081007

**Published:** 2024-08-17

**Authors:** David P. Piñero, Amparo Gil-Casas, Francisco J. Hurtado-Ceña, Ainhoa Molina-Martin

**Affiliations:** 1 Department of Optics, Pharmacology and Anatomy, University of Alicante, 03690 Alicante, Spain; 2 Department of Ophthalmology, Vithas Medimar International Hospital, 03016 Alicante, Spain; ainhoa.molina@ua.es; 3 Optometric Clinic, Lluís Alcanyís Foundation, University of Valencia, 46020 Valencia, Spain; amparo.gil@fundacions.uv.es; 4 Clínica Rementería, 28010 Madrid, Spain; javier@oftalmologiahurtado.com; 5 Instituto Nacional de la Visión SLP, 28034 Madrid, Spain

**Keywords:** amblyopia, anisometropia, dichoptic training, perceptual learning, binocular function, vision therapy

## Abstract

Objectives: This study was aimed at analyzing the efficacy on the improvement of the visual function of a dichoptic online cloud-based platform for the treatment of amblyopia in anisometropic children. Methods: A quasi-experimental (pretest–post-test) study was conducted in 23 subjects with ages from 5 to 15 years old with anisometropic amblyopia combined with additional presence (2 subjects) or not (21 subjects) of microtropia. A total of 30 home-based training sessions of 30 min per session with Bynocs^®^ platform were prescribed for 6 weeks. Results: Amblyopic eye logMAR visual acuity (VA) significantly improved from 0.28 ± 0.24 to 0.13 ± 0.20 after the 6-week treatment (*p* < 0.001). At baseline, 60.9% of participants had VA in amblyopic eye of 0.20 logMAR or worse, whereas this percentage decreased to 21.7% after treatment. Binocular function (BF) significantly improved from 2.82 ± 1.11 to 2.32 ± 0.94 (*p* < 0.001). Mean compliance was 92%, 87% and 93% at 2, 4 and 6 weeks of treatment, respectively. Conclusions: In conclusion, home-based dichoptic training with the digital platform evaluated is an effective method to improve amblyopic VA and stereoacuity in children with anisometropic amblyopia combined or not with microtropia.

## 1. Introduction

Visual training for amblyopia treatment has increased in popularity in recent years. Several software and digital platforms have been developed with the purpose of providing clinicians and subjects with different tools based on concepts such as perceptual learning and dichoptic stimulation. The use of serious games based on perceptual learning has demonstrated its usefulness on improving the visual function in subjects with amblyopia [1]. On the other hand, the use of dichoptic training has also demonstrated its usefulness as an anti-suppression treatment by presenting to the subjects different stimuli created with the purpose of balancing the visual input of each eye and improving fusion [2,3].

Conventional treatment of amblyopia has been focused on improving visual acuity (VA) on the amblyopic eye by penalizing the dominant eye through patching. This treatment has demonstrated improvements on the visual function of the amblyopic eye, but its impact on binocular function is not fully clear [4]. In the case of dichoptic training, it is focused on restabilizing the balance between eyes, since its absence has been demonstrated to be an etiological factor of suppression in amblyopia. [5]. The purpose of binocular therapies is to improve VA but combined with the treatment of binocularity, with active stimulation of binocular function [6].

A great variety of studies have demonstrated the efficacy of dichoptic training for visual function improvement in amblyopia [7,8]. In these studies, VA and other variables characterizing the visual function, such as contrast sensitivity and/or stereopsis, have been found to improve after the training, but this improvement was variable according to several factors. The variability on clinical characteristics of subjects (strabismic or anisometropic, baseline VA, age…), the type of stimuli and the mode in which they are presented to the subject (monocularly or binocularly), or even the duration of treatments (the dosage and compliance), are factors involved on the efficacy of dichoptic therapy [9].

Different videogames have been commercially released, but it is important for vision specialists to know if these serious games are effective and are really based on scientific principles [10]. The present study aims to evaluate the efficacy of visual training with a specific platform, Bynocs (Kanohi Eye Pvt. Ltd., Mumbai, India, https://www.bynocs.com/es/ (accessed on 1 March 2022)), which has been previously described and investigated by other authors [11,12,13]. These studies have demonstrated the usefulness of this platform for improving VA and binocular function in amblyopic children [12,13] and adults [13], but its efficacy has not been evaluated prospectively in a multicenter study. The aim of the current study was to provide additional evidence of the efficacy of this digital platform in children with anisometropic amblyopia or amblyopic cases associated with microtropia by evaluating the results of different Spanish centers.

## 2. Materials and Methods

### 2.1. Design

A quasi-experimental single-group pretest–post-test study.

### 2.2. Subjects

Subjects were consecutively recruited from four different Spanish eye care centers from March 2022 to July 2023: Optometric Clinic of University of Alicante (Alicante), Department of Ophthalmology of the Vithas Medimar International Hospital (Alicante), Optometric Clinic of Lluis Alcanyís Foundation of the University of Valencia (Valencia), and Clínica Rementería (Madrid).

Sample size was calculated by the online sample size calculator GRANMO (https://www.datarus.eu/aplicaciones/granmo/ (accessed on 16 August 2024)), considering the detection of a difference in VA of 0.10 logMAR, a common standard deviation of 0.11 logMAR according to a previous study [12], an alpha error of 0.05, a statistical power of 95% and a drop-out rate of 20%. A minimum sample size of 20 subjects was found to be necessary.

The study protocol was approved by the local medical Ethics Committee (Hospital General de Alicante, ISABIAL) with reference number 2021/110. All participants and their parents/guardians signed an informed consent following the tenets of Helsinki Declaration prior to inclusion as participants.

### 2.3. Selection Criteria

Inclusion criteria were anisometropic children with ages from 5 to 15 years old with amblyopia. Anisometropia was accepted as inclusion criterion when there was a difference ≥1.00 D in the spherical equivalent between eyes or a difference ≥1.50 D in the astigmatism magnitude. No restriction was applied in terms of astigmatic axes. Amblyopia was accepted as inclusion criterion when a difference of 0.10 logMAR VA was present between eyes. No exclusion criteria were applied in terms of severity of amblyopia.

Subjects could have been treated in the past with other treatments as occlusion, atropine, or binocular visual therapy with the condition of discontinuing any treatment at least 2 weeks before the inclusion in our study to avoid interferences between treatments and evaluate the effect of the proper dichoptic training. Most subjects abandoned previous treatments some months before starting, because they did not experience further improvements.

Subjects with strabismus over 10 prism diopters (PD) as measured by the prism alternating cover test were excluded, and only subjects with microstrabismus and anomalous retinal correspondence were included, that is small strabismus with some degree of binocular vision (as confirmed by the Worth 4-dot test and stereopsis measurement). Exclusion criteria were subjects with strabismus over 10 PD, nystagmus, or other ocular diseases that could affect visual performance.

Participants had to follow a training program scheduled by the visual specialist, and to assist to the control visits. In addition, participants had to be able to understand the exercises and perform the training. Since treatment was based on a home-based training, participants had to use a personal computer with internet connection, as the platform used was cloud-based.

### 2.4. Clinical Examination

Subjects were examined previously to baseline visit to ensure that they met the selection criteria. Objective and subjective refraction (with and without cycloplegia) were performed, and therefore the best refraction to prescribe was determined. Most subjects were already wearing the best refraction prior to this selection visit since they were followed by their visual specialists during the previous months (years in some cases). If any significant change in refraction was detected, refraction was changed and an adaptation period of at least 8 weeks was needed.

Baseline visit examination consisted of VA measurement in the logMAR scale with a calibrated screen optotype of letters or tumbling “E” depending on the age of the subject at far distance (4 m), heterophoria measurement by the alternating cover test and prism bar neutralization at far (6 m) and near (40 cm) distance, the Worth 4-dot test at far (6 m) and near (40 cm) distance, visuscopy to check the potential presence of eccentric fixation, and near stereopsis measurement with the TNO test (Lameris, Inc., Maryland Heights, MO, USA). The same procedures were repeated across the follow-up control visits.

The same examiner performed all clinical evaluations (baseline, 2-week, 4-week, and 6-week visits), and another examiner performed all the software measures and training, on each center.

### 2.5. Digital Platform

The digital platform used for this study was Bynocs^®^ platform (Kanohi Eye Pvt. Ltd., Mumbai, India). This cloud-based platform includes different games that can be divided into two main categories: dichoptic or binocular training games. Dichoptic training games were focused on amblyopic VA improvement though the use of dichoptic images with objects only being seen with one eye and objects being seeing by the other. The objects presented on the amblyopic eye were the key factor for the correct game performance, therefore, acting as anti-suppression elements. Binocular training games were focused on stereopsis improvement with stereograms or images with red–green disparity. Fusion and some degree of stereopsis were required to play those games.

Dichoptic stimulation was ensured with the use of red–green glasses, being the images on the screen adjusted to match the colors of the stimuli and those corresponding to the filters of the glasses to ensure that not ghosting image from the other eye was present and each type of stimulus could be properly seen by each eye. For this purpose, both the red and the green colors of the screen could be reduced in intensity, and the examiner may check with the subject for that adjustment. Before playing any game, the platform requires the measurement of some clinical parameters by the software on the platform, such as monocular far and near VA, the presence of binocularity by the Worth 4-dot test, and the measurement of heterophoria by a dissociated subjective test. If binocularity exists, then the stereopsis threshold can be measured by using a random dot stereogram. Additionally, some additional clinical data have to be introduced in the digital platform, such as interpupillary distance (IPD), which is crucial to adjust the dichoptic images and to provide the prismatic values of vergence required for some exercises. Technical aspects of the digital platform have been described in depth in a previous publication [10].

In each patient, setting of the platform characteristics on each personal computer in the baseline visit was performed by a second examiner who gave to the patient all instruction required for an appropriate use of the platform.

### 2.6. The Visual Training Procedure

Home-based visual training was scheduled for each subject during 6 weeks with a total of 30 sessions of 30 min per session. Subjects were forced to comply with 10 sessions each 2 weeks, that is 5 sessions per week with two days of rest (chosen by the subjects depending on their availability).

Subjects were instructed to play three different games per day (approx. 10 min each) during the two weeks period between evaluation visits. The games were selected depending on the clinical characteristics of each subject and their clinical evolution.

### 2.7. Statistical Analysis

With the main purpose of analyzing visual performance before and after dichoptic training, the main outcomes were best corrected VA and stereopsis, but this last parameter was analyzed by using the binocular function (BF) score [14]. BF was calculated as the logarithmic value of the stereopsis in sec arc, and it was scored as 4 in the case of fusion or diplopia, and 5 in the case of suppression as measured by the Worth 4-dot test. Clinical measures were assessed before and after (2 weeks, 4 weeks and 6 weeks) the training. Additionally, information about compliance of the training was also collected.

Statistical analysis was performed with the SPSS software v28.0 (IBM statistics, Armonk, NY, USA). Normality was assessed with the Kolmogorov–Smirnov test, which revealed that most data distributions used were non-normally distributed. Therefore, non-parametric tests were applied. Statistical significance of differences between consecutive visits were assessed with the Friedman test, whereas the post hoc comparison between pair of visits was assessed by the Wilcoxon test with the Bonferroni adjustment. A *p*-value of 0.05 was considered statistically significant.

## 3. Results

### 3.1. Sample Characteristics

A total of 23 subjects (10 males and 13 females) from 5 to 15 years old (mean value 9 ± 3 years) were included. All included subjects performed the prescribed sessions and attended all the follow-up visits. None of the subjects referred to diplopia during the treatment, or reported any other relevant visual disturbance. Interpupillary distance (IPD) varied from 52 to 63 mm (mean value 57 ± 3 mm). Both exophoric (negative values) and esophoric (positive values) subjects were included with a mean value of 0.22 ± 5.39 (from −16 to 14 PD) for far and 0.28 ± 7.39 PD (from −18 to 14 PD) for near distance. Subjects with microtropia and anomalous retinal correspondence represented a total of 9% of the sample (2 of 23 participants). Most subjects (20 of 23) were patients previously treated by their visual specialists for months before recruitment, with perfect optical adaptation and some previous unsuccessful treatments. A total of 70% of subjects (16 of 23) received previous treatment with patching (4 of them combined patch with visual therapy), and 17% (4 of 23) were considered too old for patching when detected.

Eyes were divided by dominant eyes and amblyopic eyes. The spherical equivalent (SE) of dominant eyes vary between 5.50 and −2.38 D (mean value 1.76 ± 1.92 D) and in amblyopic eyes from 19 to −21.50 D (mean value 3.26 ± 6.77 D). A high percentage of the participants showed hyperopia (21 of 23 of the subjects). Half of participants (12 of 23 of the subjects) showed a low or moderate anisometropia (difference between eyes ≤ 2D), and the rest showed a severe anisometropia. Three subjects had astigmatic anisometropia, and nine subjects presented a combination of astigmatic and spherical anisometropia.

### 3.2. Clinical Measures

Visual parameters were measured on four visits (baseline, 2 weeks, 4 weeks and 6 weeks): VA (logMAR) of the dominant eye, VA of the amblyopic eye (logMAR), and BF (in log sec arc). Results were summarized in Table 1.

VA showed statistically significant differences between visits for both dominant (*p* = 0.009) and amblyopic (*p* < 0.001) eyes. In both cases, a decrease in the mean logMAR values, that is an improvement in VA, was observed across visits. The difference between eyes was significantly reduced across visits (*p* < 0.001). In addition, BF showed a statistically significant decrease, that is a significant improvement on stereopsis across the visits (*p* < 0.001). No statistically significant differences between visits were found for the cover test for far (baseline 0.22 ± 5.39 PD; 2 weeks 0.47 ± 4.73 PD; 4 weeks 0.29 ± 4.00 PD; 6 weeks 0.00 ± 4.34 PD; *p* = 0.307) and near (baseline 0.22 ± 5.39 PD; 2 weeks 0.12 ± 7.28 PD; 4 weeks 0.06 ± 7.30 PD; 6 weeks 0.28 ± 6.72 PD; *p* = 0.990) distance.

#### 3.2.1. Visual Acuity

**Figure 1:**
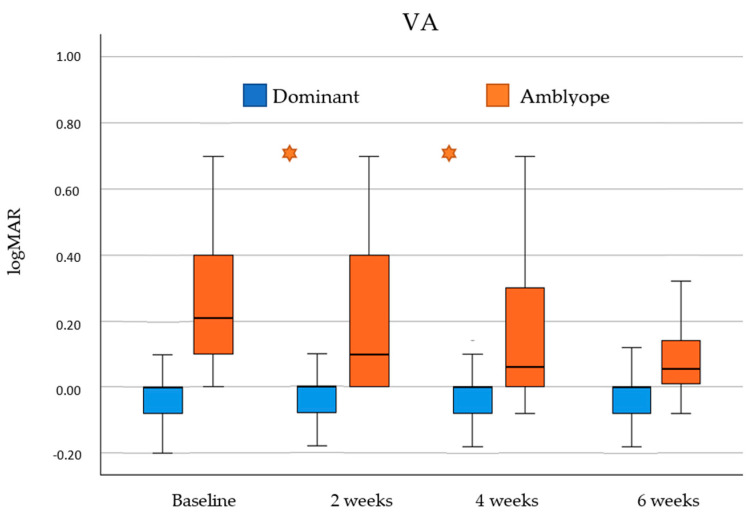
Visual acuity (VA) results obtained across visits of this study (baseline, 2 weeks, 4 weeks and 6 weeks) for dominant (blue) and amblyopic (orange) eyes. Statistically significant differences between continuous visits were marked with an asterisk (*).

VA improvement was analyzed in dominant and amblyopic eyes across the four visits of this study (baseline, 2 weeks, 4 weeks and 6 weeks). Differences between consecutive visits were analyzed and results are showed in Figure 1.

Comparison between baseline and final visit for dominant eyes showed a statistically significant increase in VA (*p* = 0.013). However, this increase was not statistically significant between baseline and the 2-week visit (*p* = 0.307), between the 2-week and 4-week visit (*p* = 0.058), and between the 4-week and 6-week visits (*p* = 0.343).

Comparison between baseline and final visit for amblyopic eyes showed a statistically significant improvement in VA (*p* < 0.001). Additionally, this improvement was more pronounced and statistically significant in the first month of treatment, that is between baseline and 2-week visits (*p* < 0.001) and between 2-week and 4-week visits (*p* = 0.011). This improvement was not statistically significant between 4-week and 6-week visits (*p* = 0.221).

At baseline, 60.9% of participants (14 of 23) had a VA in the amblyopic eye of 0.20 logMAR or worse, and 87% of participants (20 of 23) had a VA of 0.10 logMAR or worse. After the six-week treatment, 21.7% of the participants (5 of 23) had VA in the amblyopic eye of 0.20 logMAR or worse, and 47.8% of participants (11 of 23) a VA of 0.1 logMAR or worse.

#### 3.2.2. Binocular Function

**Figure 2:**
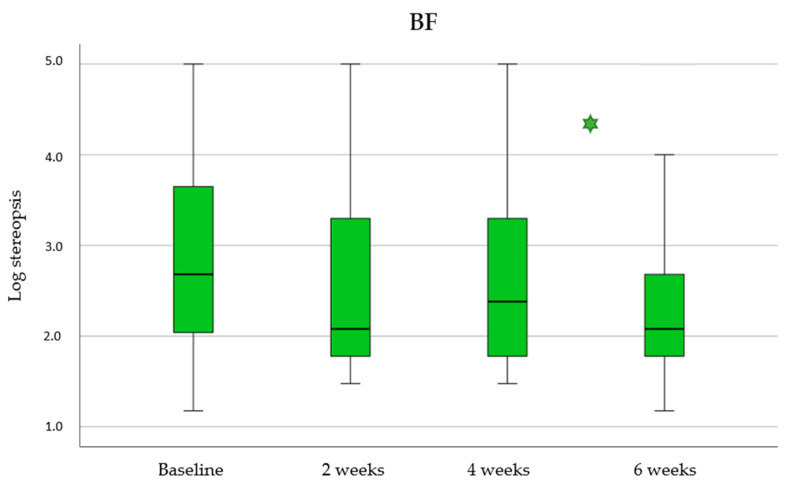
Binocular Function (BF) results obtained across visits of this study (baseline, 2 weeks, 4 weeks and 6 weeks). Statistically significant differences between continuous visits were marked with an asterisk (*).

BF improvement was analyzed across the four visits of this study (baseline, 2 weeks, 4 weeks and 6 weeks). Differences between consecutive visits were analyzed and results are showed in Figure 2.

Comparison between baseline and final visit for BF showed a statistically significant decrease in the score (*p* < 0.001) and therefore a significant improvement in stereopsis. Additionally, this improvement was more pronounced and statistically significant in the last two weeks of treatment, that is between the 4-week and the 6-week visits (*p* = 0.006). This increase was not statistically significant between baseline and the 2-week visits (*p* = 0.378) and either between the 2-week and 4-week visits (*p* = 0.419).

At baseline, 74% of participants (17 of 23) achieved stereopsis and only 39% a value better (less) than 120 sec arcs. After the six-week treatment, 91% of the participants (21 of 23) achieved measurable stereopsis by TNO (1980 sec arc or less) and 57% achieved a value <120 sec arc (13 of 23), and 48% values ≤60 sec arc (11 of 23).

### 3.3. Compliance

All included subjects performed 6 weeks of treatment and attended the four intermediate visits (at baseline, 2 weeks, 4 weeks and 6 weeks). Participants were encouraged to train 30 min per day, with sessions 5 days per week as indicated by the software. According to this, 10 sessions across two weeks of training for 6 weeks should be performed by the subjects. A total of 15 h of training was programed per patient (30 session × 30 min).

Number of sessions performed during the two weeks period between visits was recorded as a measure of compliance of the treatment by the participants. Number of sessions (mean value ± SD) was 9.2 ± 1.6 (6 to 11) during the first 2 weeks of treatment, 8.7 ± 2.2 (3 to 10) during the second period of weeks of treatment, and 9.3 ± 1.2 (6 to 10) in the last two weeks of treatment. Total number of sessions performed by the participants (mean value ± SD) was 27.0 ± 4.5 (16 to 30).

In terms of percentage of compliance, mean percentage of compliance at 2-week visit was 92% (percentage of the total time performed in that period, that is 9.2 sessions completed out of10 that should be performed), at 4-week visit was 87% and at 6-week visit was 93%.

First period sessions, comprising the first two weeks of training between baseline and the 2-week visit, were completed (10 sessions across the two weeks) by 70% of participants. The rest of the participants (7 of 23) performed between 7 and 9 sessions. Second period sessions, comprising the period between the 2-week and 4-week visits, were completed by 65% of participants. The rest of the participants (8 of 23) performed between 3 and 9 sessions. Third period sessions, comprising the last two weeks of training, were also completed by 65% of participants. The rest of the participants (8 of 23) performed from 6 to 9 sessions.

## 4. Discussion

Current visual screening campaigns developed by public services and vision specialists during childhood have increased the early detection of refractive problems, leading to a decrease in the prevalence of amblyopia due to optical correction. A high percentage of subjects with amblyopia due to anisometropia is recovered only with optical correction as treatment, but there is a residual group of subjects in which even a conventional treatment with patching fails [4,6,8]. These subjects need an additional therapeutic option to obtain a recovery of binocular function, being dichoptic therapy great help for this purpose. Binocular training with serious games in a dichoptic environment has demonstrated to induce improvements in VA and binocular function of subjects with amblyopia [6,7,8]. Based on the current scientific evidence, the purpose of the present study was to analyze the efficacy of a new digital platform in terms of improvement of the visual function of anisometropic children with and without microtropia after a 6-week period of home-based training. For this purpose, the sample included both myopic and hyperopic subjects of different magnitudes from 5 to 15 years old. In this sample, most subjects were detected and optically corrected on time and received a previous treatment with patching in the past, but a residual amblyopia was still present when enrolled in our trial.

The level of amblyopia was not considered as a selection criterion, but the sample was limited to low and mild amblyopic subjects, with some cases presenting only one logMAR VA line of difference between eyes. Therefore, the possibility of improvement was limited following the treatment, since it has been demonstrated that subjects with poorer VA baseline values tend to show better levels of VA improvements with dichoptic therapy [15]. This is the case of the study of Abdal et al. [12], who analyzed retrospectively the visual function improvements of a sample of 127 anisometropic children between 4 and 13 years old. These authors used the same digital platform as in our study and followed a similar protocol, finding a mean improvement of approximately 0.40 logMAR in VA after 6 weeks of dichoptic treatment. Compared to our sample, the study of Abdal et al. [12] included younger children without previous treatments and a poorer baseline VA (0.59 logMAR vs. 0.28 logMAR in this case) that were recruited in the early stages of the detection (only 21% was previously treated). Picotti et al. [13] also found a mean improvement with dichoptic treatment using the Bynocs platform of 0.30 logMAR in a sample of anisometropic children between 8 and 16 years old with a mean baseline VA value of 0.46 logMAR. In these two previous studies evaluating the digital platform Bynocs, subjects with severe amblyopia were included.

Our sample was comprised of anisometropic subjects, but the sample was not totally pure since some of the included subjects showed a small angle of strabismus with some degree of gross binocular vision. These subjects were included with the purpose of reinforcing binocularity since it has been demonstrated that dichoptic training can be also useful in strabismic subjects [16], but in the present study, the sample was limited to small angles with anomalous retinal correspondence to avoid large suppression scotomas and allowing the stabilization of the fragile anomalous binocularity of this type of patients. Heterogeneity of the sample could be another limitation for improvements on visual function of the present study, since improvements are more limited in the presence of strabismus [15], requiring higher durations of treatment for binocular restoration and the combination with other treatments options such as surgery, prismatic correction or spherical additions [17]. Even if there was no evident improvement in VA, dichoptic training induces improvements in other aspects of the visual function [18,19]. In the present study, BF also showed improvements following the treatment, changing its mean value from 2.82 to 2.32, with 9% of stereo deficient subjects after the treatment compared to the 26% that was present before the treatment. This improvement was smaller than in previous studies using the same dichoptic platform and similar protocols [12,13], but in that case, the baseline BF values of the samples were worse than those present in the present sample. Regarding the cover test analysis, this was not a main outcome of the analysis in the present study, but the subjective ocular deviation was recorded in all visits to monitor the subject evolution considering that some subjects had microstrabismus. The purpose of the treatment was not supposed to affect the orthoptics of the eye, and effectively no significant changes in the level of ocular deviation were found after the treatment, but this aspect was considered because some games of the platform were focused on vergence training.

A great advantage of the treatment by a gamified software for home-based training purposes is the level of stimulation of attention achieved, with the additional motivation of a scoring system in which the subjects can track his/her own evolution. Serious games have demonstrated better compliance than conventional therapies such as patching [20,21], but they are not exempt of limitations in terms of age (small children) and loss of interest [21,22,23] depending on the game. Additionally, the recording of the game parameters and subjects’ responses provides an additional control over compliance that now can be totally monitored by the online platforms. In the present study, subjects were instructed to play 30 min of game per day, 5 days a week for 6 weeks. Thus, the total training duration was 15 h. This time was less than the treatment durations from other protocols, but it was enough to produce changes on the visual function as suggested by the present results and those obtained previously with the same digital platform [12,13]. Compared with other studies in which compliance was evaluated with patching [20,21,23,24], our results showed greater compliance, with values over 85%. In any case, it should be considered that the sample was comprised of subjects treated in the past with residual amblyopia, and therefore the motivation of subjects for finding something to improve was very high. It is important to note that this home-based dichoptic training was supervised online by a visual specialist, which additionally performed control visits every two weeks with the purpose of adapting the games to the evolution in subject clinical characteristics, but also to maintain the motivation and a closer follow-up of the subjects. This could also provoke the higher levels of compliance recorded compared with other protocols. In any case, it should be considered that patient engagement with therapy may be related to many factors, such as the level of intuitiveness of the game, if it is fun, the level of adaptation of the game to the preferences of the children which are age related, the level of interaction required by the game, or even the children’s emotional status [25]. In this study, all these factors could not be monitored and some of them may have contributed to the high compliance found.

Main limitations of the current study are the lack of measurements of contrast sensitivity to investigate further the impact on the visual function, the lack of measurements of patient-reported outcomes by means of validated questionnaires to confirm the impact perceived by the patient of the treatment, the lack of ongoing follow-up after completing 6 weeks of treatment to ensure that visual gains are stable in a longer term, and not including a control group for comparison. Future comparative studies should be performed with patients treated with patching and patients treated with other dichoptic or visual training validated procedures.

## 5. Conclusions

Home-based dichoptic training with the digital platform evaluated can be an effective method to improve amblyopic VA and stereoacuity in children with residual amblyopia. Future studies should be conducted to investigate if the effect obtained with this fast and easy to perform visual training is maintained in the long term and if a longer duration of the treatment could lead to still better outcomes (only 6 weeks of treatments were prescribed). Likewise, the impact of the use of this digital platform in strabismic amblyopic subjects in combination with other therapeutic options (surgery, prism or addition) should be investigated further.

## Figures and Tables

**Table 1:** Summary of the mean values ± SD (minimum to maximum) of the measured visual parameters across the four visits (baseline, 2 weeks, 4 weeks and 6 weeks). Abbreviations: VA, visual acuity; BF, binocular function.

Clinical	Baseline	2 Weeks	4 Weeks	6 Weeks
VA dominant	0.00 ± 0.09 −0.20 to 0.22	−0.02 ± 0.10 −0.18 to 0.22	−0.03 ± 0.10 −0.26 to 0.15	−0.04 ± 0.10 −0.26 to 0.12
VA amblyopic	0.28 ±0.24 0.00 to 1.00	0.20 ± 0.24 0.00 to 0.70	0.15 ± 0.21 −0.08 to 0.70	0.13 ± 0.20 −0.08 to 0.70
BF	2.82 ± 1.11 1.18 to 5.00	2.62 ± 0.98 1.48 to 5.00	2.61 ± 0.96 1.48 to 5.00	2.32 ± 0.94 1.18 to 5.00

## Data Availability

Dataset available on request from the authors.
